# Dietary supplementation with Chinese herb ultrafine powder improves intestinal morphology and physical barrier function by altering jejunal microbiota in laying hens

**DOI:** 10.3389/fmicb.2023.1185806

**Published:** 2023-05-16

**Authors:** Jue Gui, Md Abul Kalam Azad, Wenchao Lin, Chengwen Meng, Xin Hu, Yadong Cui, Wei Lan, Jianhua He, Xiangfeng Kong

**Affiliations:** ^1^Key Laboratory of Agro-ecological Processes in Subtropical Region, Hunan Provincial Key Laboratory of Animal Nutrition Physiology and Metabolic Processes, Institute of Subtropical Agriculture, Chinese Academy of Sciences, Changsha, Hunan, China; ^2^School of Biology and Food Engineering, Fuyang Normal University, Fuyang, Anhui, China; ^3^College of Animal Science and Technology, Hunan Agricultural University, Changsha, Hunan, China

**Keywords:** Chinese herb, intestinal barrier function, microbiota, ultrafine powder, Xinyang black-feather hens

## Abstract

**Introduction:**

Chinese medicinal herbs play important roles in anti-inflammatory, antioxidant, and antibacterial activities. However, the effects of Chinese herb ultrafine powder (CHUP) on laying hens still need to be elucidated. Therefore, this study aimed to evaluate the effects of dietary CHUP supplementation on jejunal morphology, physical barrier function, and microbiota in laying hens.

**Methods:**

A total of 576 Xinyang black-feather laying hens (300 days old) were randomly assigned into eight groups, with eight replicates per group and nine hens per replicate. The hens were fed a basal diet (control group) and a basal diet supplemented with 0.5% *Leonuri herba* (LH group), 0.25% *Ligustri lucidi fructus* (LF group), 0.25% *Taraxaci herba* (TH group), 0.5% LH + 0.25% LF (LH-LF group), 0.5% LH + 0.25% TH (LH-TH group), 0.25% LF + 0.25% TH (LF-TH group), and 0.5% LH + 0.25% LF + 0.25% TH (LH-LF-TH group), respectively, for 120 days.

**Results:**

The results showed that dietary LH-LF and LH-LF-TH supplementation increased (*p* < 0.05) the jejunal villus height to crypt depth ratio of laying hens. Dietary LF-TH supplementation up-regulated jejunal *claudin-5* expression, while LH supplementation up-regulated jejunal *claudin-1* expression and increased the jejunal abundances of potentially beneficial bacteria related to short-chain fatty acids and bacteriocins production, such as *Blautia*, *Carnobacterium*, *Clostridiales*, and *Erysipelotrichales* (*p* < 0.05). In addition, dietary LH supplementation enriched (*p* < 0.05) the tetracycline biosynthesis, butirosin/neomycin biosynthesis, and D-arginine/D-ornithine metabolism, whereas steroid biosynthesis and limonene/pinene degradation were enriched (*p* < 0.05) in the LH-LF and LH-LF-TH groups. Moreover, Spearman’s correlation analysis revealed the potential correlation between the abundance of the jejunal microbiota and jejunal morphology and the physical barrier function of laying hens.

**Discussion:**

Collectively, these findings suggest that dietary CHUP supplementation could enhance the beneficial bacteria abundance, physical barrier function, and metabolic function associated with short-chain fatty acids and bacteriocins production. Moreover, combined supplementation of dietary CHUP showed better effects than the sole CHUP supplementation.

## Introduction

Laying hens during the late laying phase are characterized by decreased laying performance, egg quality, and an imbalanced immune system (Ma et al., 2014). In addition, intestinal lipid metabolism, energy production, and antioxidant metabolism are also affected during the late laying phase of laying hens (Wang et al., 2019). It is well-known that gut microbiota plays important roles in overall host health (Shu et al., 2011). Intestinal microbiota not only regulates digestion and absorption of nutrients (Chang and Martinez-Guryn, 2019) but also influences immune homeostasis and chronic diseases (Gao et al., 2018). In addition, the intestinal microbiota of high-yield laying hens could increase the egg-laying rates of low-yield laying hens by modulating the relative abundance of intestinal microbiota (Wang et al., 2020). Previous studies also reported that the enhancement of intestinal mucosal barrier functions could increase egg production by enhancing the immunity of the intestinal tissue in laying hens (Nii, 2022). Thus, maintaining a healthy intestinal development is critical for improving the overall health of laying hens, as disorders of the intestinal microbiota have several adverse effects. Therefore, it is necessary to explore the effects of feed additives on laying hens that can improve laying performance and egg quality by maintaining intestinal barrier integrity and microbiota balance.

Feed additives are commonly used to improve animal health and productivity and inhibit toxins in the intestine to promote nutrient absorption (Ashour et al., 2020). Antibiotics, a type of traditional feed additives, are banned in most countries due to the major threat to public health caused by antibiotic resistance (Huang et al., 2018). In the recent few decades, various antibiotic alternatives, such as natural polyamines (Ma et al., 2020), amino acids (Dong et al., 2017), organic acids (Gong et al., 2021), vitamins (Li et al., 2022), probiotics (Abd El-Hack et al., 2020), and Chinese herbs and their extracts (Cheng et al., 2014) have been used to improve the production performance and product quality in poultry. These feed additives could improve animal health by transplantation of spermidine-altered microbiota (Ma et al., 2020), producing intestinal mucin (Cheng et al., 2014), and altering the selection pressures on the resident microbiota (Bedford and Cowieson, 2012). Meanwhile, Chinese herbs have shown various beneficial effects, such as anti-inflammation, antioxidant, and anti-bacterial properties (Han et al., 2019), which further influence the gut microbiota structure and microbiota-mediated bioconversion (Chen et al., 2016). In addition, Chinese herbs were also found to modulate intestinal barrier function and improve metabolic endotoxemia (Zhang et al., 2021). Moreover, Chinese herbs could promote the production of intestinal short-chain fatty acids (SCFAs) by increasing the abundance of *Roseburia* and *Eubacterium* (Zhang et al., 2019), and thereby showed ideal anti-inflammatory effects by increasing the abundance of anti-inflammatory associated gut bacteria (Wei et al., 2018). Therefore, Chinese herbs might have the potential to be served as a prophylactic or therapeutic way to affect gut barrier integrity and intestinal microbiota. However, to date, few studies have investigated the interplays between Chinese herbs and the intestinal microbiota of laying hens.

According to the medical experience from a traditional Chinese medical doctor and the theory of “sovereign, minister, assistant, and courier”, *Leonuri herba* (LH), *Ligustri lucidi fructus* (LF), and *Taraxaci herba* (TH) design appropriate dose to prepare Chinese herb ultrafine powder (CHUP). The LH is a sovereign medicine that activates the blood to regulate menstruation, heat-clearing, and detoxification, therefore, its dosage is larger than that of LF and TH. The LF showed better effects on the liver and kidney, while TH could effectively reduce swelling and disperse stagnation (Chinese Pharmacopoeia Commission, 2020). Moreover, LF and TH have been used together as minister medicine since one was a tonifying and replenishing medicine, and the other was a heat-clearing medicine. In recent years, ultrafine grinding technology has been applied to develop novel feed formulations. Compared with the traditional decoction pieces, due to the advantage of smaller particle size, CHUP showed improved dissolution of the active ingredients of drugs by increasing the damage rate of the plant cell walls, thereby improving the bioavailability of Chinese herbs in humans (Rao et al., 2019).

We hypothesized that supplementing Chinese herbs in laying hens diet during the late laying phase might positively stimulate the overall health of laying hens by influencing the intestinal morphology and altering the microbial community composition. Hence, to test these hypotheses, this study investigated the effects of dietary CHUP supplementation on the intestinal health of the laying hens, which might provide a feasible nutritional and low-cost dietary formulation approach to promote intestinal health and extend the laying period of laying hens.

## Materials and methods

### Experimental birds, diets, and management

A total of 576 healthy 300 days old Xinyang black-feather laying hens were randomly assigned into eight groups. Each group contained eight replicates with nine hens per replicate. After 7 days of the adaptation period, the birds in the control group were fed a basal diet, and the birds in the treatment groups were fed a basal diet supplemented with 0.5% *Leonuri herba* (LH group), 0.25% *Ligustri lucidi fructus* (LF group), 0.25% *Taraxaci herba* (TH group), 0.5% LH + 0.25% LF (LH-LF group), 0.5% LH + 0.25% TH (LH-TH group), 0.25% LF + 0.25% TH (LF-TH group), and 0.5% LH + 0.25% LF + 0.25% TH (LH-LF-TH group), respectively. The CHUP (particle size distribution, D95 < 48 μm) additives were provided by Kangxin*g* Pharmaceutical Co., Ltd. (Henan, China). The experiment lasted 120 days. Three hens were housed per cage, kept in four-layer ladder cages, and had free access to feed and drinking water at all times. The birds were housed in a room with a 16 h/8 h light/dark cycle and fed twice daily at 07:30 and 16:00 h with their respective diets throughout the experimental period; water was available by a nipple-type drinker. The room temperature was maintained at approximately 25°C. According to the layer feeding standard (NY/T 33-2004), the basal diet was formulated based on the nutritional requirements during the late laying period (Table 1).

**Table 1:** Ingredients and nutritional composition of the basal diet, % air-dried.

Ingredients	Content	Nutrient component^b^	Content
Corn	62.59	Metabolizable energy (MJ/kg)	11.25
Soybean meal	23.88	Crude protein	14.23
Limestone powder	7.94	Ether extract	9.66
Soybean oil	0.49	Calcium	3.51
Methionine	0.10	Phosphorus	0.34
Premix^a^	5.00	Lysine	0.71
Total	100.00	Methionine	0.37

a The ingredients provided per kilogram of premix: vitamin A, 140,000 IU; vitamin D_3_, 50,000 IU; vitamin E, 480 mg; vitamin Κ3, 0.18 g; vitamin B_1_, 63 mg; vitamin B_2_, 200 mg; vitamin B_6_, 140 mg; vitamin B_12_, 0.7 mg; nicotinic acid, 1,000 mg; D-pantothenic acid, 500 mg; folic acid, 50 mg; D-biotin, 5.0 mg; choline chloride, 900 mg; Fe, 2.0 g; Cu, 0.3 g; Mn, 1.8 g; Zn, 2.0 g; I, 70 mg; Se, 9 mg; and phytase, 3,000 IU.

b Crude protein and ether extract were measured values, while the others were calculated values.

### Sample collection

At the end of the experiment (days 120 of the trial), eight laying hens were selected for euthanization from each group. The middle sections of the jejunum (2 cm in length) were collected and then fixed in 10% neutral-buffered formalin for morphological analysis. Jejunal (middle position) contents were collected into sterile tubes, immediately frozen into liquid nitrogen, and stored at −80°C for intestinal microbiota analysis. The jejunal tissues were collected into RNase-free tubes, frozen into liquid nitrogen, and stored at −80°C for gene expression analysis.

### Histomorphological observation

The fixed segments of the jejunal tissues were dehydrated, clarified, and embedded in paraffin. Slides were selected from the sections (approximately 5-μm thickness), stained with hematoxylin-eosin (HE), and finally sealed. The villus height (VH, from the tip of the villus to the villus-crypt junction) and crypt depth (CD, from the crypt opening to the base) of the jejunum were determined using an optical microscope (Nikon Eclipse CI, Tokyo, Japan) with image-processing software. The VH to CD ratio (VCR) was then calculated.

### Gene expression analysis related to jejunal physical barrier function

The total RNA of the jejunal tissues was extracted using the AG RNAex Pro reagent (Accurate Biology, Changsha, China). The purity and concentration of the extracted total RNA were determined using a NanoDrop ND-2000 spectrophotometer (Thermo Fischer Scientific, Waltham, MA, US). The total RNA (1 μg) was reversely transcribed into cDNA using the PrimeScript RT Reagent Kit with gDNA Eraser (Accurate Biology) for quantitative PCR analysis. The real-time quantitative PCR (RT-qPCR) was performed on the Light Cycler^®^ 480 II Real-Time PCR System (Roche, Basel, Switzerland). Primer sequences were synthesized by the Tsingke Biotechnology Co., Ltd. (Beijing, China) and used in this study (listed in Supplementary Table S1). The RT-qPCR was carried out in a 10-μL reaction system, consisting of 4.2 μl of cDNA template, 0.4 μl of each primer, and 5.0 μl SYBR^®^ Green mix (AG, Changsha, China). The RT-qPCR conditions were as follows: an initial step at 95°C for 5 min, followed by 40 cycles of denaturation at 95°C for 5 s and annealing at 60°C for 30 s, and a final extension at 72°C for 30 s. The relative mRNA expression levels were calculated using β-actin as the internal control and the 2^−ΔΔCt^ method (Rao et al., 2013).

### Jejunal microbiota analysis

The microbial DNA from the individual jejunal contents (200 mg) was extracted using the QIAamp DNA Stool Mini Kit (Qiagen, Hilden, Germany) according to the manufacturer’s instructions. The V3–V4 regions of the 16S rRNA gene were amplified using PCR with primers 338F (5′-ACTCCTACGGGAGGCAGCA-3′) and 806R (5′-GGACTACHVGGGTWTCTAAT-3′). The PCR reaction conditions were as follows: an initial denaturation at 98°C for 2 min, followed by 25 cycles of denaturation at 95°C for 15 s and annealing at 55°C for 30 s, extension at 72°C for 30 s, and a final extension at 72°C for 5 min. PCR amplified products were recovered by 1.20% Agarose Gel Extraction Kit (Invitrogen, Carlsbad, CA, US) and quantified using the Quant-iT PicoGreen dsDNA Assay Kit (Invitrogen, Carlsbad, CA, US). Illumina MiSeq sequencing and bioinformatics analysis were performed by the Shanghai Personal Biotechnology Co., Ltd. (Shanghai, China) on the Illumina NovaSeqPE 250 platform (Illumina, San Diego, CA, US). Sequences were processed and taxonomy was assigned using the Quantitative Insights into Microbial Ecology 2 (QIIME2). Amplicon sequence variants (ASVs) were determined with DADA2 by denoising. The taxonomic annotation of ASVs was implemented using the Greengenes database. Alpha diversity indices (including Observed_species richness, Shannon index, Faith’s Phylogenetic Diversity, and Pielo’s Evenness index) were determined to evaluate the richness and evenness of microbial species. Beta diversity was evaluated by principal coordinate analysis (PCoA) based on the Jaccard distance. Taxonomic distributions at the phylum, family, and genus levels were statistically compared based on the results of taxonomic annotation among different treatment groups. Linear discriminant analysis (LDA) combined effect size (LEfSe) was performed to explore the biomarkers in the relative abundances of bacteria among different groups using Wilcoxon’s test. Spearman’s correlation analysis (*R* > 0.5, *p* < 0.05) among the top 50 phyla, families, and genera was conducted to determine the potential relationship between jejunal morphology, gene expression level, and microbial composition.

### Statistical analysis

After the test data were initially organized by Excel, one-way ANOVA was performed using the SPSS (version 26.0) software, and Tukey’s *post-hoc* test was performed to compare the statistical significance among different treatment groups. Data are presented as means and standard error of mean. *p* value < 0.05 was considered for statistical significance.

## Results

### Effects of dietary Chinese herb ultrafine powder on jejunal morphology of laying hens

**Figure 1:**
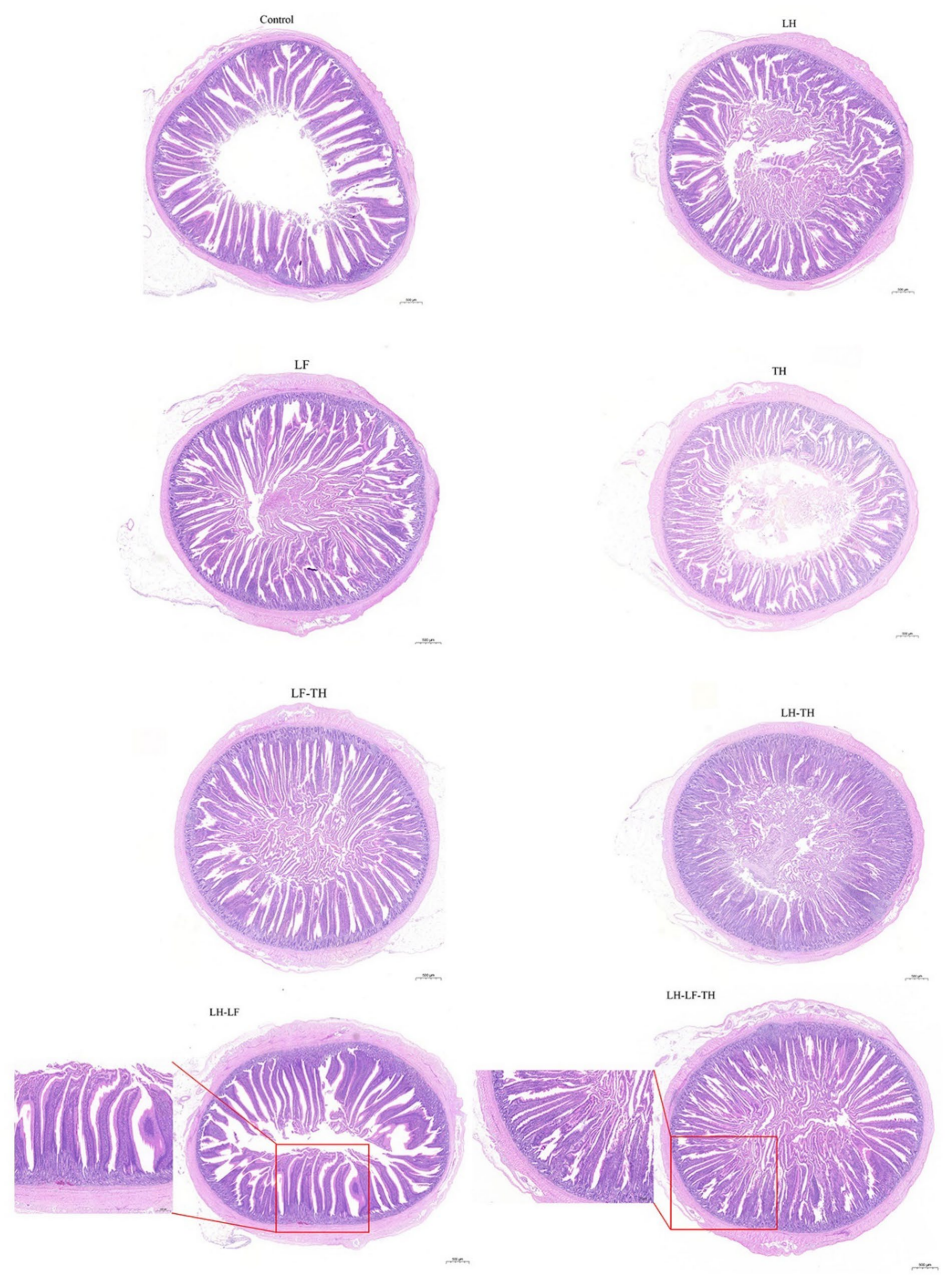
Effects of dietary Chinese herb ultrafine powder supplementation on jejunal morphology of laying hens (HE staining). Scale bars, 500 μm (magnification 20×). Control group, fed a basal diet; LH group, basal diet supplemented with 0.5% *Leonuri herba*; LF group, basal diet supplemented with 0.25% *Ligustri lucidi fructus*; TH group, basal diet supplemented with 0.25% *Taraxaci herba*; LH-LF group, basal diet supplemented with 0.5% LH + 0.25% LF; LH-TH group, basal diet supplemented with 0.5% LH + 0.25% TH; LF-TH group, basal diet supplemented with 0.25% LF + 0.25% TH; LH-LF-TH group, basal diet supplemented with 0.5% LH + 0.25% LF + 0.25% TH.

The effects of dietary CHUP supplementation on the jejunal morphology of laying hens are presented in Table 2 and Figure 1. The jejunal VH was lower (*p* < 0.05) in the TH and LH-TH groups compared with the control and other treatment groups (except the LF group). In addition, jejunal VH was higher (*p* < 0.05) in the LH-LF group compared with the other treatment groups (except the LH group). The jejunal VCR was higher (*p* < 0.05) in the LH-LF and LH-LF-TF groups compared with the LH-TH group. However, dietary CHUP supplementation did not affect (*p* > 0.05) the jejunal CD of laying hens (Table 2). Moreover, there were no visible differences in the jejunal morphology of laying hens (Figure 1).

**Table 2:** Effects of dietary Chinese herb ultrafine powder supplementation on jejunal morphology of laying hens.

Items	Dietary groups								SEM	*p*-values
	Control	LH	LF	TH	LH-LF	LH-TH	LF-TH	LH-LF-TH		
VH (μm)	1074.48^ab^	1078.23^ab^	1025.55^bc^	970.04^c^	1138.09^a^	967.09^c^	1049.19^b^	1066.15^b^	8.537	<0.001
CD (μm)	240.54	233.39	194.05	213.71	194.86	244.23	212.39	188.50	6.191	0.126
VCR	4.55^ab^	5.16^ab^	5.31^ab^	4.74^ab^	6.05^a^	4.07^b^	5.09^ab^	5.94^a^	0.153	0.011

Data are expressed as means with their SEM (*n* = 8). Means within a row without a common superscript letter are significantly different (*p* < 0.05). VH, villus height; CD, crypt depth; VCR, VH to CD ratio; Control group, fed a basal diet; LH group, basal diet supplemented with 0.5% *Leonuri herba*; LF group, basal diet supplemented with 0.25% *Ligustri lucidi fructus*; TH group, basal diet supplemented with 0.25% *Taraxaci herba*; LH-LF group, basal diet supplemented with 0.5% LH + 0.25% LF; LH-TH group, basal diet supplemented with 0.5% LH + 0.25% TH; LF-TH group, basal diet supplemented with 0.25% LF + 0.25% TH; LH-LF-TH group, basal diet supplemented with 0.5% LH + 0.25% LF + 0.25% TH.

### Effects of dietary Chinese herb ultrafine powder on gene expressions related to jejunal physical barrier functions in laying hens

**Figure 2:**
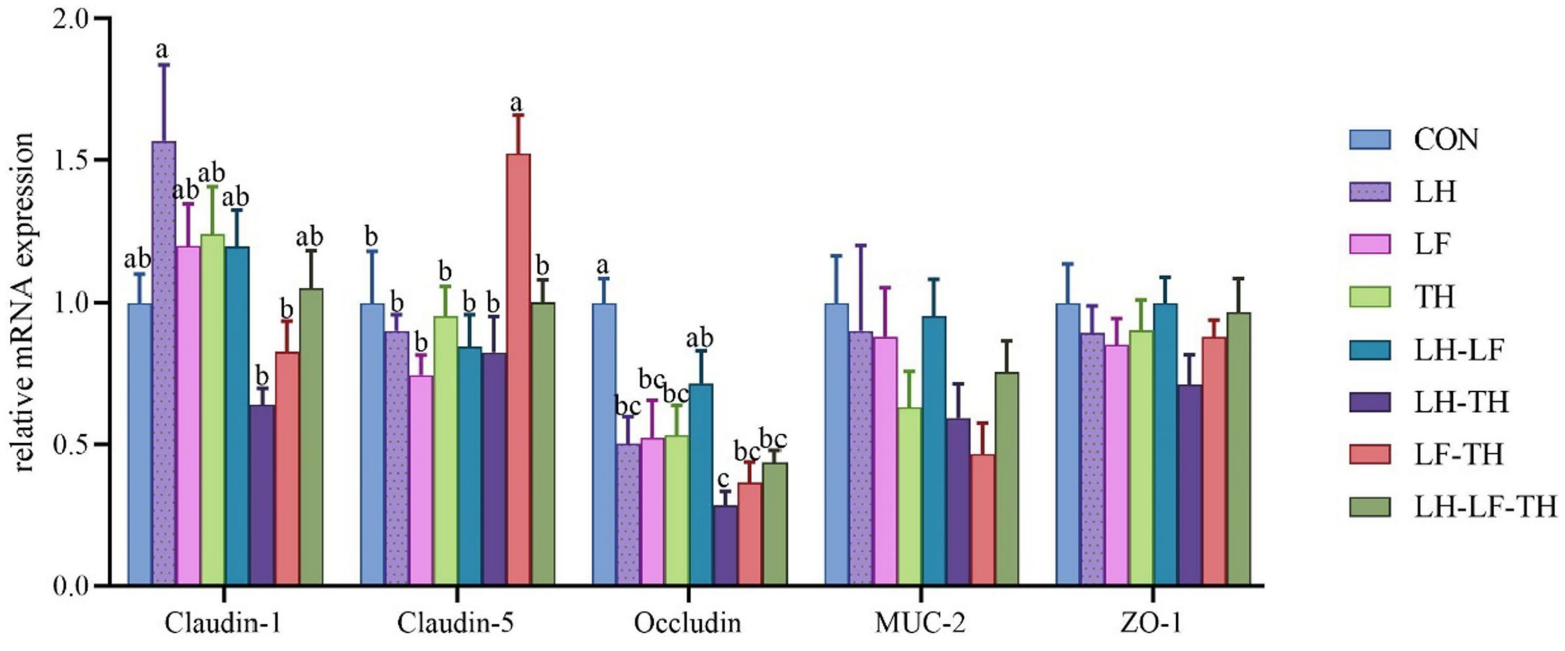
Effects of dietary Chinese herb ultrafine powder supplementation on physical barrier function-related gene expressions in the jejunum of laying hens (*n* = 8). ^a–c^ Different superscript letters in the histogram indicate significant differences among different groups (*p* < 0.05). *MUC-2*, mucin-2; *ZO-1*, zonula occludens-1. CON, control group, fed a basal diet; LH group, basal diet supplemented with 0.5% *Leonuri herba*; LF group, basal diet supplemented with 0.25% *Ligustri lucidi fructus*; TH group, basal diet supplemented with 0.25% *Taraxaci herba*; LH-LF group, basal diet supplemented with 0.5% LH + 0.25% LF; LH-TH group, basal diet supplemented with 0.5% LH + 0.25% TH; LF-TH group, basal diet supplemented with 0.25% LF + 0.25% TH; LH-LF-TH group, basal diet supplemented with 0.5% LH + 0.25% LF + 0.25% TH.

The gene expressions related to the jejunal physical barrier function of different treatment groups are shown in Figure 2. Dietary LH-TH and LF-TH supplementation down-regulated (*p* < 0.05) the jejunal *claudin-1* expression compared with the LH group. In addition, dietary LF-TH supplementation up-regulated (*p* < 0.05) the jejunal *claudin-5* expression compared with the other groups. Moreover, jejunal *occludin* expression was down-regulated (*p* < 0.05) in all treatment groups (except the LH group) compared with the control group. However, no significant differences (*p* > 0.05) were observed in mucin-2 (*MUC-2*) and zonula occludens-1 (*ZO-1*) expressions in the jejunum of laying hens in response to dietary CHUP supplementation.

### Effects of dietary Chinese herb ultrafine powder on jejunal microbiota profile in laying hens

**Figure 3:**
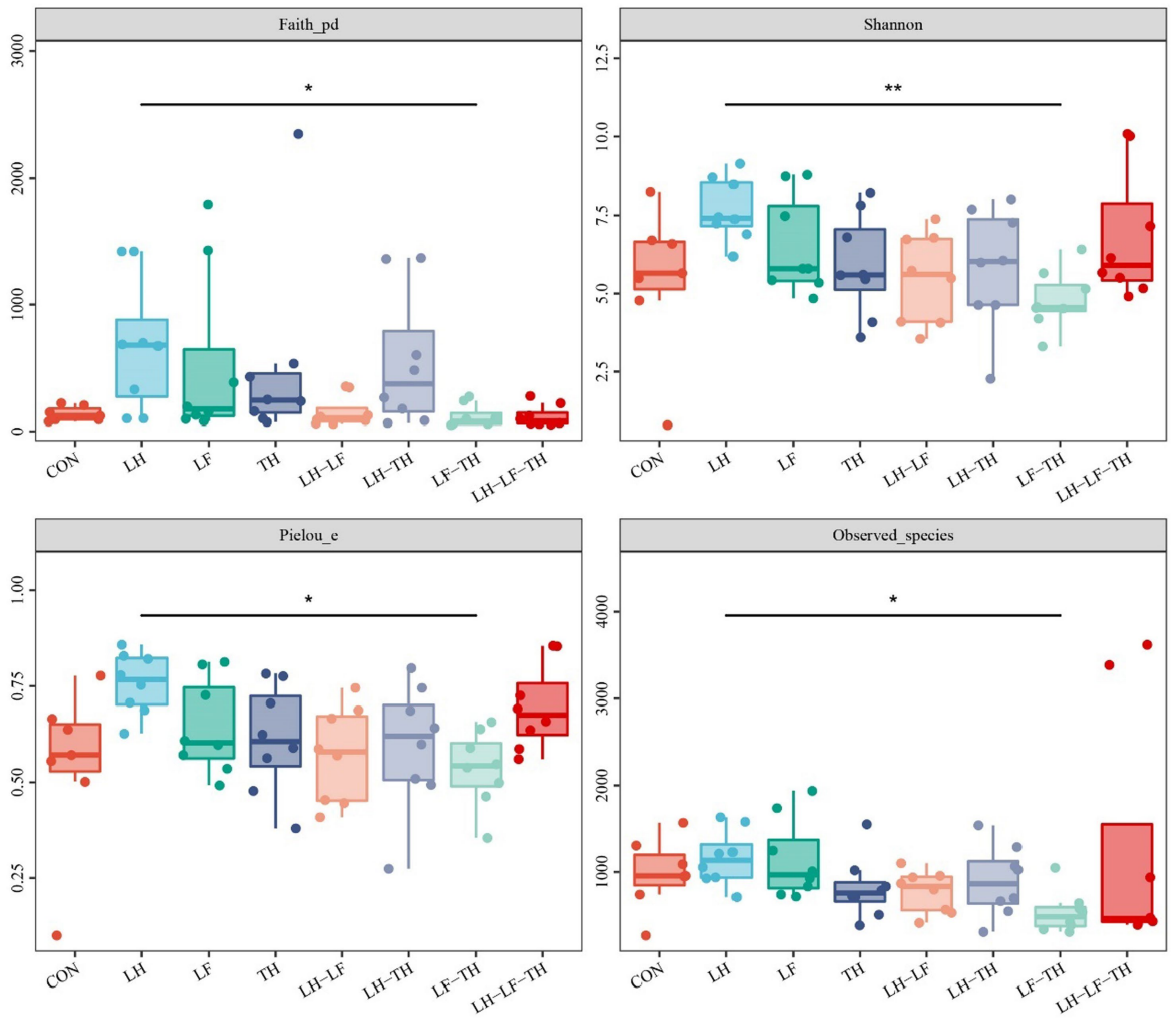
Effects of dietary Chinese herb ultrafine powder supplementation on alpha diversity indices of jejunal microbiota of laying hens (*n* = 7–8). *p < 0.05, **p < 0.01. CON, control group, fed a basal diet; LH group, basal diet supplemented with 0.5% *Leonuri herba*; LF group, basal diet supplemented with 0.25% *Ligustri lucidi fructus*; TH group, basal diet supplemented with 0.25% *Taraxaci herba*; LH-LF group, basal diet supplemented with 0.5% LH + 0.25% LF; LH-TH group, basal diet supplemented with 0.5% LH + 0.25% TH; LF-TH group, basal diet supplemented with 0.25% LF + 0.25% TH; LH-LF-TH group, basal diet supplemented with 0.5% LH + 0.25% LF + 0.25% TH.

**Figure 4:**
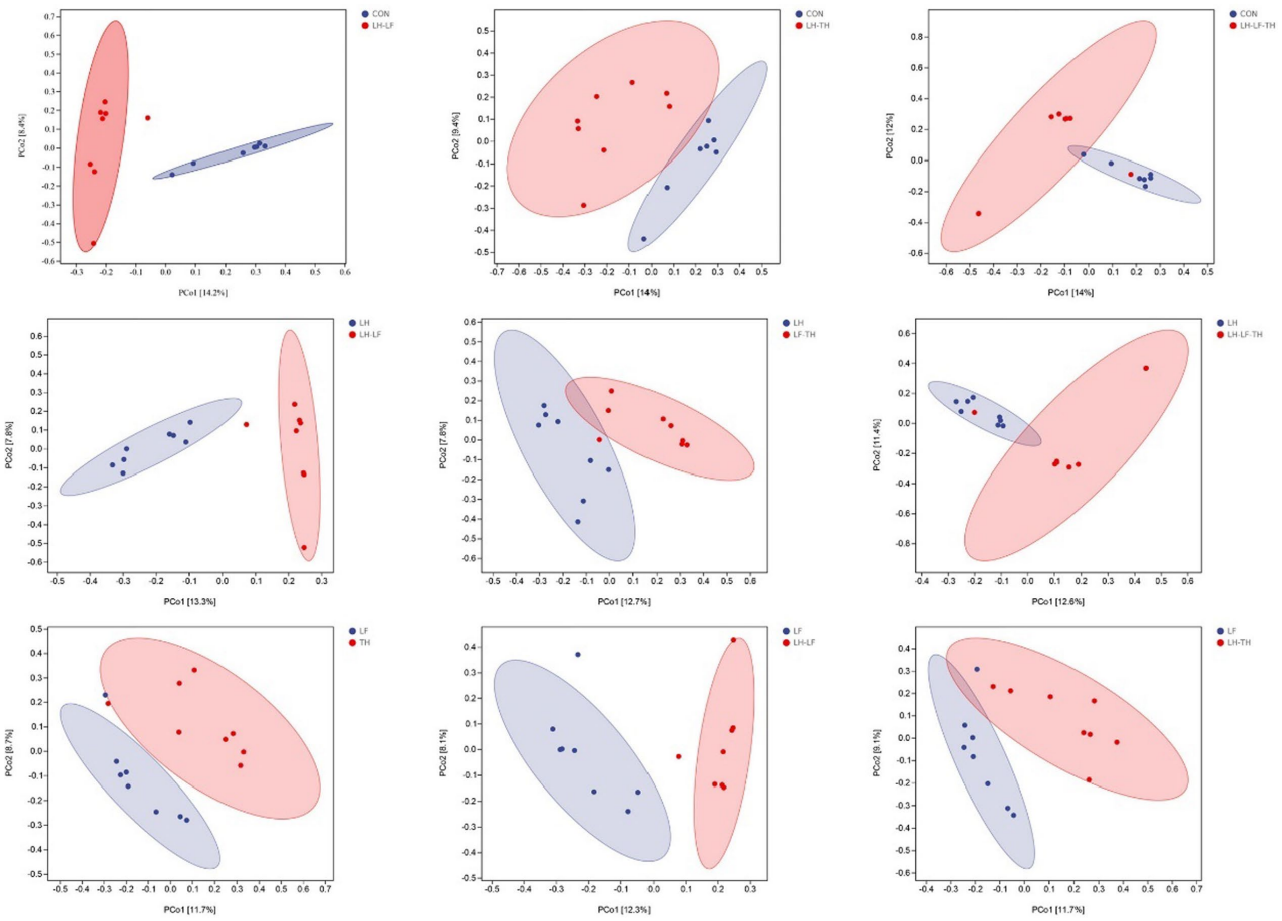
Effects of dietary Chinese herb ultrafine powder supplementation on β-diversity of jejunal microbiota of laying hens (*n* = 7–8). Principal coordinate analysis (PCoA) of microbiota based on Jaccard distances. CON, control group, fed a basal diet; LH group, basal diet supplemented with 0.5% *Leonuri herba*; LF group, basal diet supplemented with 0.25% *Ligustri lucidi fructus*; TH group, basal diet supplemented with 0.25% *Taraxaci herba*; LH-LF group, basal diet supplemented with 0.5% LH + 0.25% LF; LH-TH group, basal diet supplemented with 0.5% LH + 0.25% TH; LF-TH group, basal diet supplemented with 0.25% LF + 0.25% TH; LH-LF-TH group, basal diet supplemented with 0.5% LH + 0.25% LF + 0.25% TH.

A total of 7,988,670 valid sequences were obtained from 64 samples of the jejunal contents in laying hens using the DADA2 plugin. Alpha diversity analysis was conducted to evaluate the diversity and structure of the jejunal microbiota communities in different dietary treatments. As shown in Figure 3, no significant differences (*p* > 0.05) were observed between the treatment and control groups; however, there were distinct separations (*p* < 0.05) between the LH and LF-TH groups in Observed_species, Faith’s PD, Pielou_e, and Simpson indices. As shown in Figure 4, the PCoA analysis revealed that there were significant separations between the control and LH-LF, LH-TH, or LH-LF-TH groups.

**Figure 5:**
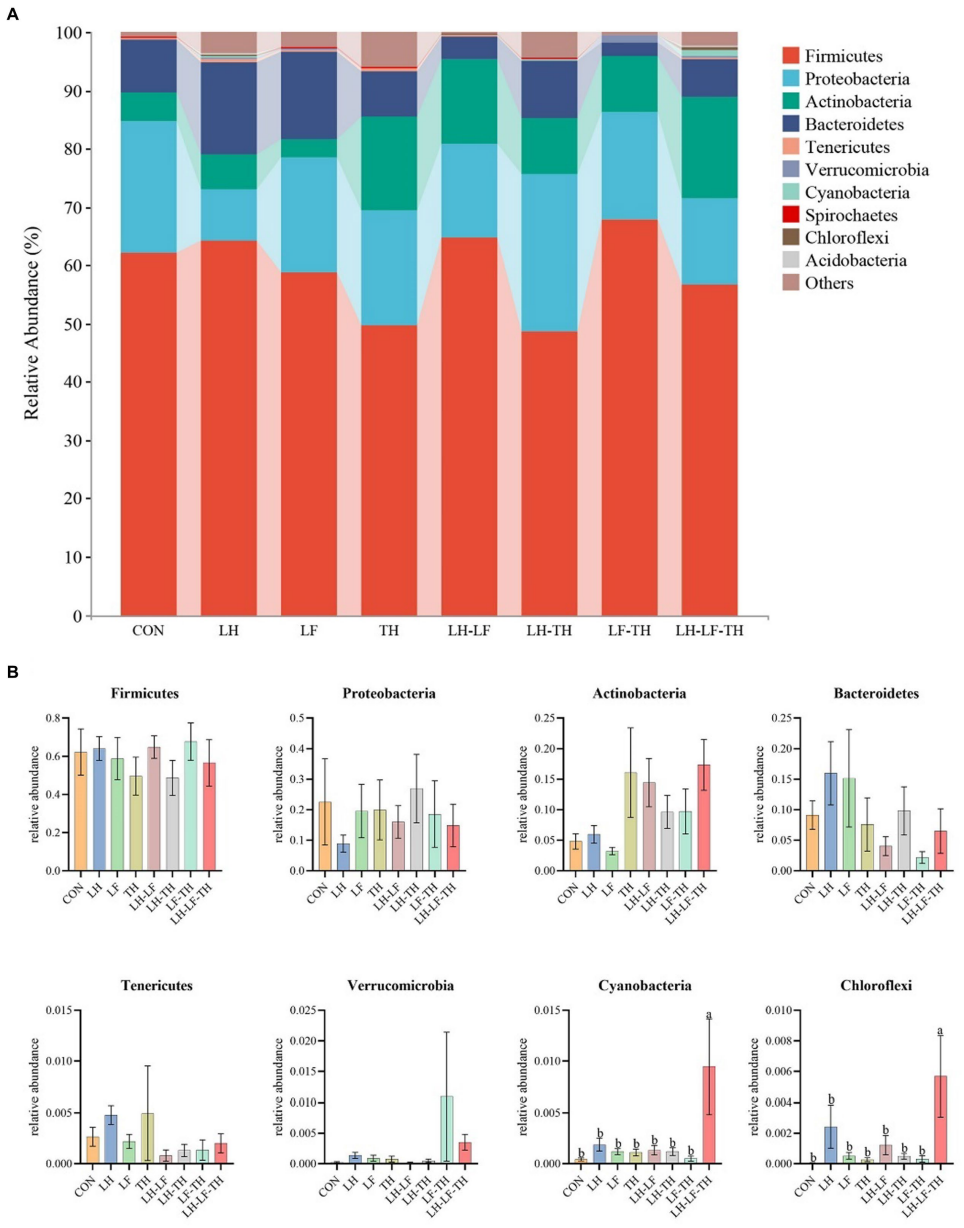
Effects of dietary Chinese herb ultrafine powder supplementation on microbial community composition in the jejunum of laying hens at the phylum level (*n* = 7–8). **(A)** Taxonomic composition at the phylum level, and **(B)** taxonomic differences among different groups. ^a,b^ Different superscript letters in the histogram indicate significant differences among different groups (*p* < 0.05). CON, control group, fed a basal diet; LH group, basal diet supplemented with 0.5% *Leonuri herba*; LF group, basal diet supplemented with 0.25% *Ligustri lucidi fructus*; TH group, basal diet supplemented with 0.25% *Taraxaci herba*; LH-LF group, basal diet supplemented with 0.5% LH + 0.25% LF; LH-TH group, basal diet supplemented with 0.5% LH + 0.25% TH; LF-TH group, basal diet supplemented with 0.25% LF + 0.25% TH; LH-LF-TH group, basal diet supplemented with 0.5% LH + 0.25% LF + 0.25% TH.

**Figure 6:**
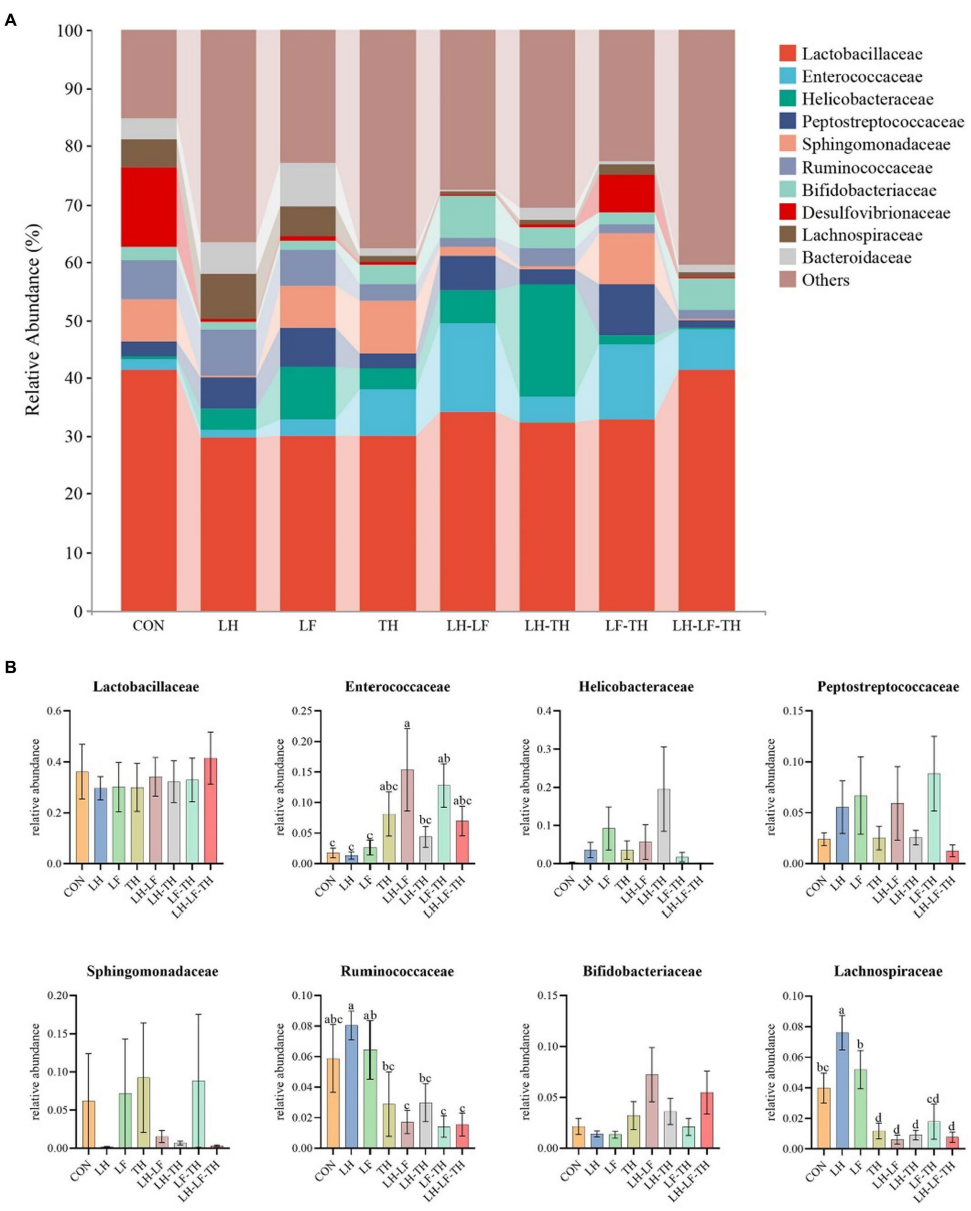
Effects of dietary Chinese herb ultrafine powder supplementation on microbial community composition in the jejunum of laying hens at the family level (*n* = 7–8). **(A)** Taxonomic composition at the family level, and **(B)** taxonomic differences among different groups. ^a–d^ Different superscript letters in the histogram indicate significant differences among different groups (*p* < 0.05). CON, control group, fed a basal diet; LH group, basal diet supplemented with 0.5% *Leonuri herba*; LF group, basal diet supplemented with 0.25% *Ligustri lucidi fructus*; TH group, basal diet supplemented with 0.25% *Taraxaci herba*; LH-LF group, basal diet supplemented with 0.5% LH + 0.25% LF; LH-TH group, basal diet supplemented with 0.5% LH + 0.25% TH; LF-TH group, basal diet supplemented with 0.25% LF + 0.25% TH; LH-LF-TH group, basal diet supplemented with 0.5% LH + 0.25% LF + 0.25% TH.

**Figure 7:**
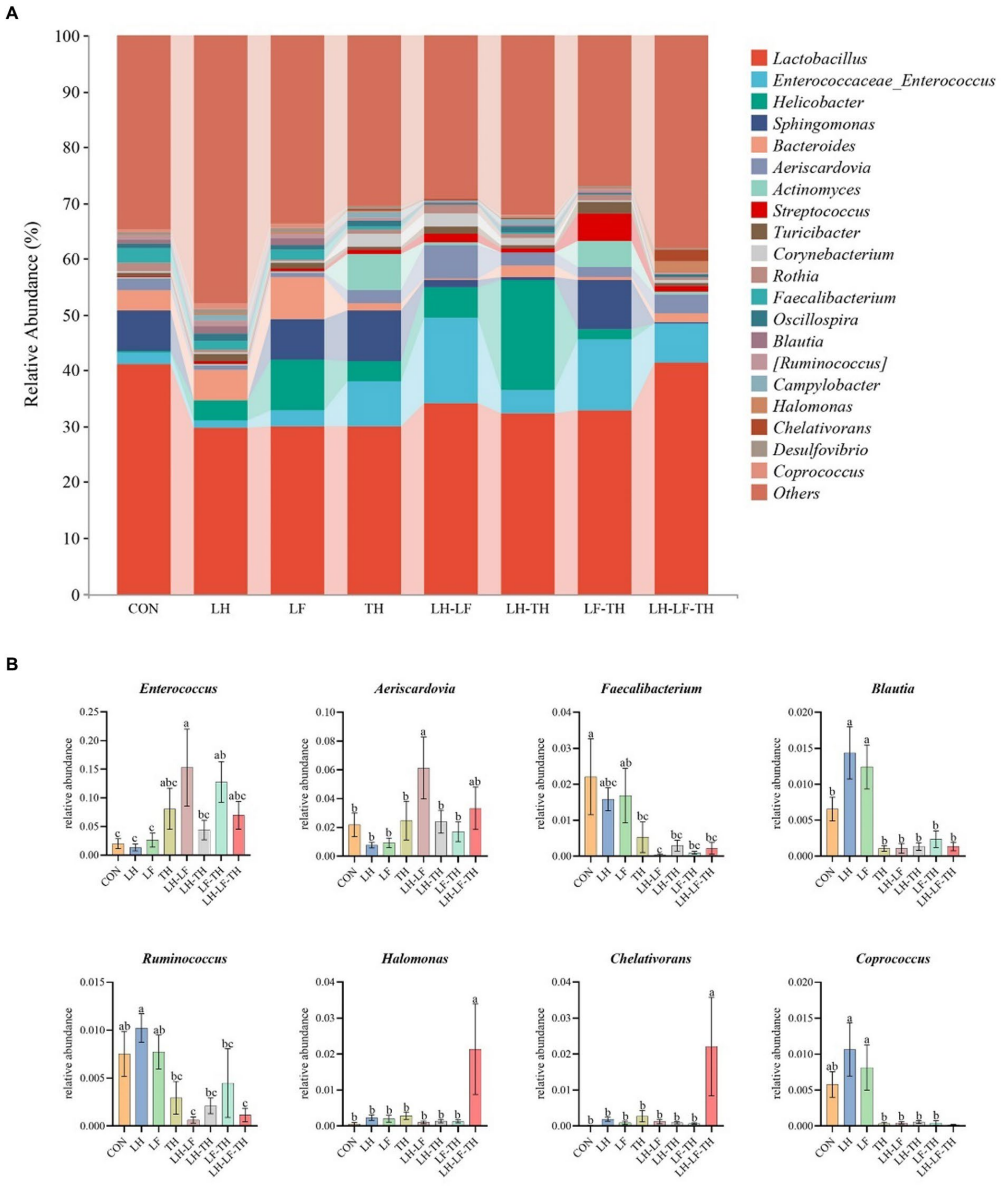
Effects of dietary Chinese herb ultrafine powder supplementation on microbial community composition in the jejunum of laying hens at the genus levels (*n* = 7–8). **(A)** Taxonomic composition at the genus levels, and **(B)** taxonomic differences among different groups. ^a–c^ Different superscript letters in the histogram indicate significant differences among different groups (*p* < 0.05). CON, control group, fed a basal diet; LH group, basal diet supplemented with 0.5% *Leonuri herba*; LF group, basal diet supplemented with 0.25% *Ligustri lucidi fructus*; TH group, basal diet supplemented with 0.25% *Taraxaci herba*; LH-LF group, basal diet supplemented with 0.5% LH + 0.25% LF; LH-TH group, basal diet supplemented with 0.5% LH + 0.25% TH; LF-TH group, basal diet supplemented with 0.25% LF + 0.25% TH; LH-LF-TH group, basal diet supplemented with 0.5% LH + 0.25% LF + 0.25% TH.

The jejunal microbiota composition was analyzed at the phylum (Figure 5), family (Figure 6), and genus (Figure 7) levels. At the phylum level (Figure 5A), *Firmicutes* (59.07%) and *Proteobacteria* (18.41%) were the most dominant phyla, followed by *Actinobacteria* (10.14%), *Bacteroidetes* (8.79%), *Tenericutes* (0.25%), *Verrucomicrobia* (0.23%), and others (3.12%). The relative abundances of *Cyanobacteria* and *Chloroflexi* were higher (*p* < 0.05) in the LH-LF-TH group compared with the other groups (Figure 5B). At the family level (Figure 6A), *Lactobacillaceae* (33.97%) and *Enterococcaceae* (6.70%) were the most dominant families, followed by *Helicobacteraceae* (5.45%), *Peptostreptococcaceae* (4.51%), *Sphingomonadaceae* (4.38%), *Ruminococcaceae* (3.97%), and others (41.01%). The relative abundance of *Enterococcaceae* was higher (*p* < 0.05) in the LH-LF and LF-TH groups, as well as *Lachnospiraceae* in the LH group, when compared with the control group. However, the abundance of *Lachnospiraceae* in the TH, LH-LF, LH-TH, and LH-LF-TH groups was lower (*p* < 0.05) compared with the control group (Figure 6B). At the genus level (Figure 7A), *Lactobacillus* (33.92%) and *Enterococcus* (6.69%) were the two most dominant phyla, followed by *Helicobacter* (5.45%), *Sphingomonas* (4.30%), *Bacteroides* (2.74%), *Aeriscardovia* (2.49%), and others (44.41%). The relative abundances of *Enterococcus* in the LH-LF and LF-TH groups, *Aeriscardovia* in the LH-LF group, *Chelativorans* and *Halomonas* in the LH-LF-TH group, and *Blautia* in the LH and LF groups were higher (*p* < 0.05) compared with the control group. The relative abundance of *Faecalibacterium* in the LH-LF group was lower (*p* < 0.05) compared with the control and LF groups, whereas *Ruminococcus* was lower (*p* < 0.05) in the LH-LF and LH-LF-TH groups compared with the control group (Figure 7B).

**Figure 8:**
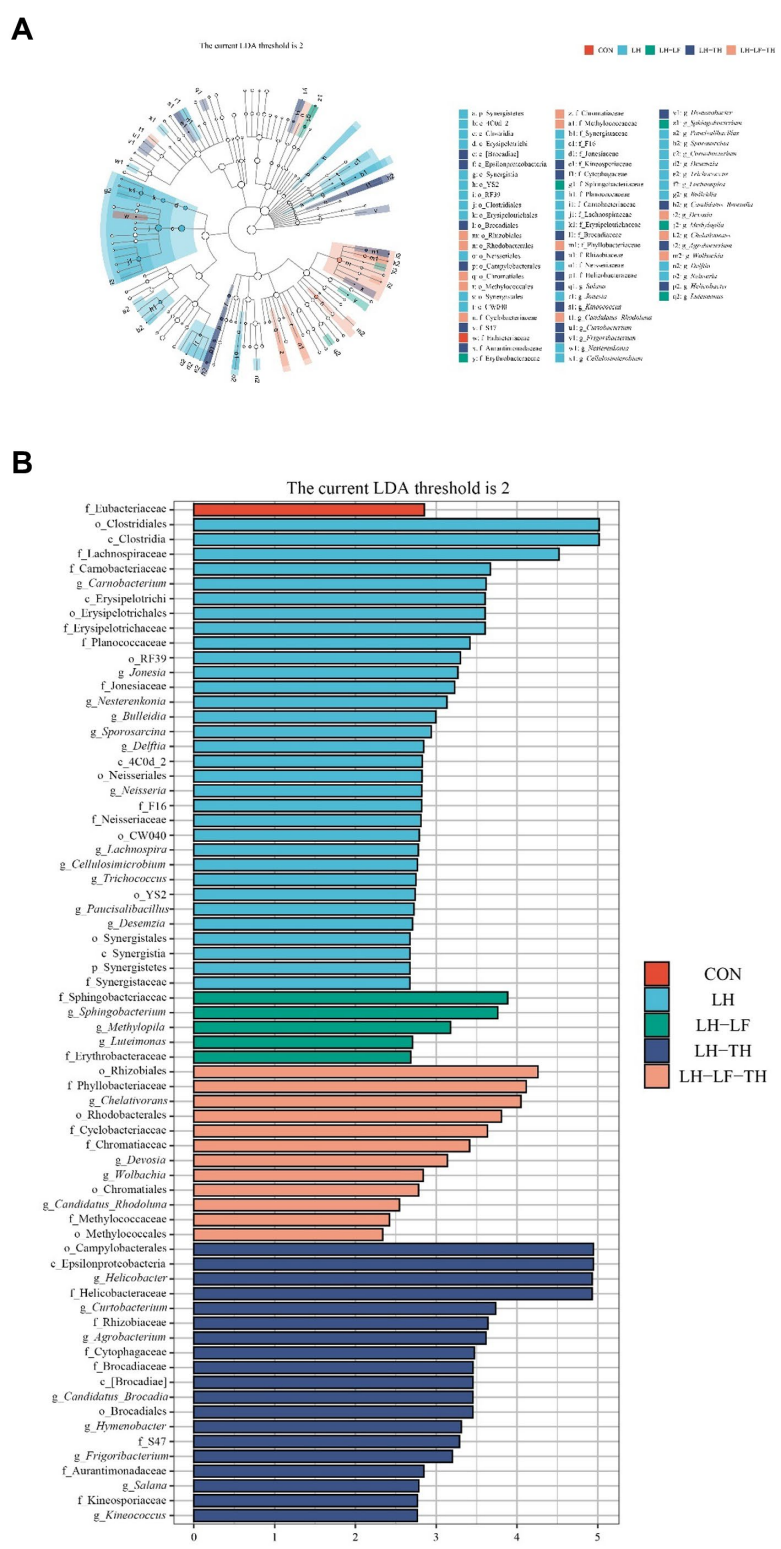
Effects of dietary Chinese herb ultrafine powder supplementation on differential bacterial taxa in the jejunum of laying hens (*n* = 7–8). **(A)** Taxonomic cladogram and **(B)** histogram of the distribution of LDA values for different species identified by the LEfSe algorithm. Species with significant difference have an LDA score greater than the estimated value (2.0). CON, control group, fed a basal diet; LH group, basal diet supplemented with 0.5% *Leonuri herba*; LF group, basal diet supplemented with 0.25% *Ligustri lucidi fructus*; TH group, basal diet supplemented with 0.25% *Taraxaci herba*; LH-LF group, basal diet supplemented with 0.5% LH + 0.25% LF; LH-TH group, basal diet supplemented with 0.5% LH + 0.25% TH; LF-TH group, basal diet supplemented with 0.25% LF + 0.25% TH; LH-LF-TH group, basal diet supplemented with 0.5% LH + 0.25% LF + 0.25% TH.

The LEfSe analysis (Figures 8A,B) revealed that 32 bacterial biomarkers were enriched (LDA threshold > 2) in the jejunum of the LH group, including (LDA > 4) *Clostridiales*, *Clostridia*, and *Lachnospiraceae*. The abundances of *Springobacteriaceae, Sphingobacterium*, *Methylopila*, *Luteimonsa*, and *Erythrobacteraceae* were enriched in the LH-LF group. In the LH-LF-TH group, 12 bacterial biomarkers were enriched (LDA > 2) in the jejunum of laying hens, including (LDA > 4) *Rhizobiales*, *Phyllobacteriaceae*, and *Chelativorans*. Moreover, 19 bacterial biomarkers were enriched (LDA > 2) in the jejunum of the LH-TH group, including (LDA > 4) *Camphylobacerales*, *Epsilonproteobacteria*, *Helicobacter*, and *Helicobacteraceae*. However, only *Eubacteriaceae* was enriched (LDA > 2) in the control group.

### Effects of dietary Chinese herb ultrafine powder on function prediction of jejunal microbiota in laying hens

**Figure 9:**
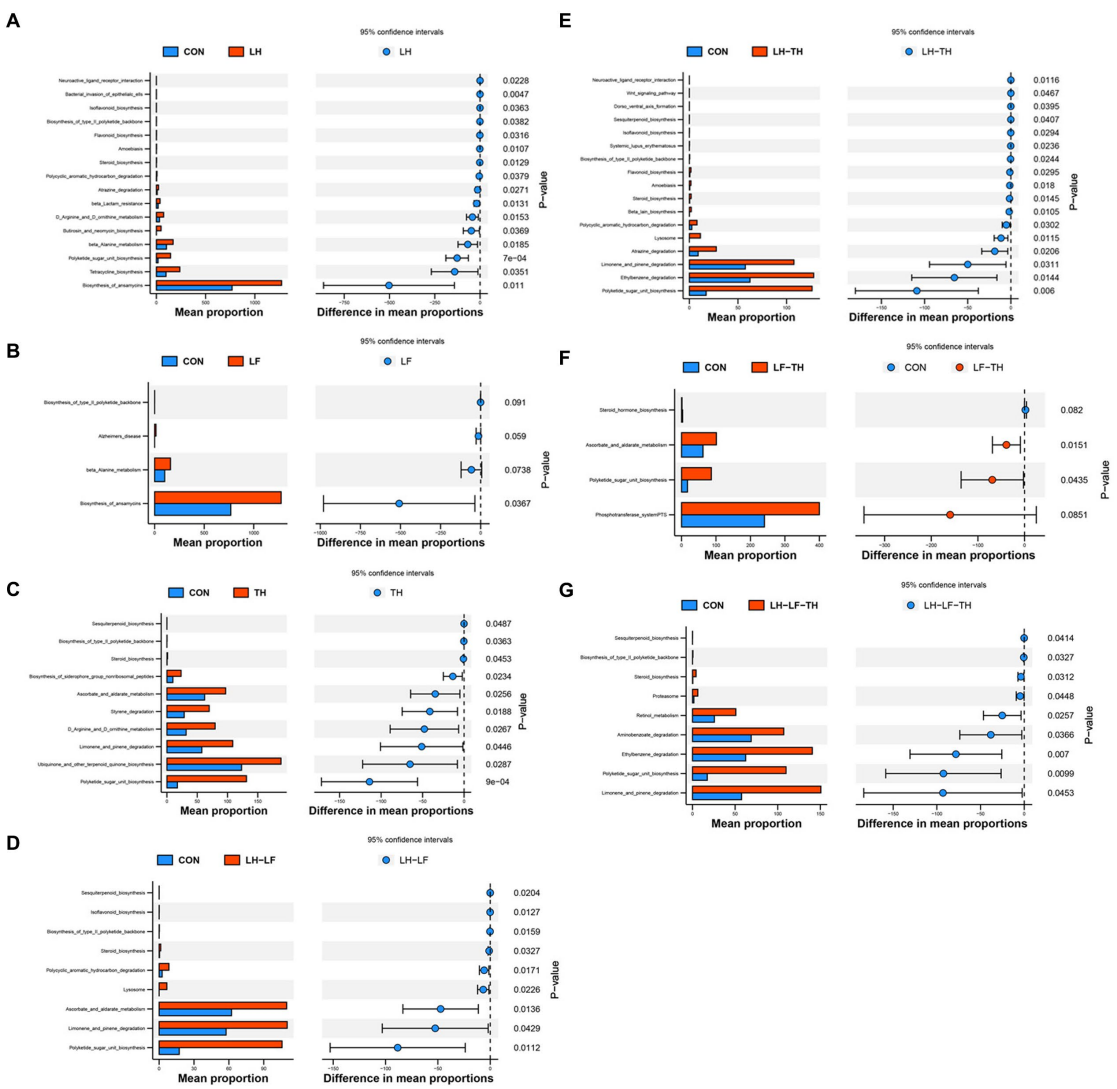
Differences in metabolic functions based on PICRUSt2 analysis of jejunal microbiota of laying hens. **(A)** CON vs. LH, **(B)** CON vs. LF, **(C)** CON vs. TH, **(D)** CON vs. LH-LF, **(E)** CON vs. LH-TH, **(F)** CON vs. LF-TH, and **(G)** CON vs. LH-LF-TH. CON, control group, fed a basal diet; LH group, basal diet supplemented with 0.5% *Leonuri herba*; LF group, basal diet supplemented with 0.25% *Ligustri lucidi fructus*; TH group, basal diet supplemented with 0.25% *Taraxaci herba*; LH-LF group, basal diet supplemented with 0.5% LH + 0.25% LF; LH-TH group, basal diet supplemented with 0.5% LH + 0.25% TH; LF-TH group, basal diet supplemented with 0.25% LF + 0.25% TH; LH-LF-TH group, basal diet supplemented with 0.5% LH + 0.25% LF + 0.25% TH.

The functional abundances of jejunal microbial communities predicted by the PICRUSt2 are shown in Figure 9. Dietary LH supplementation increased (*p* < 0.05) the relative abundances of microbial genes involved in the ansamycins, tetracycline biosynthesis, β-lactam resistance, β-alanine metabolism, butirosin and neomycin biosynthesis, and polyketide sugar unit biosynthesis compared with the control group (Figure 9A). Dietary LF supplementation increased (*p* < 0.05) the relative abundances of microbial genes involved in the biosynthesis of ansamycins compared with the control group (Figure 9B). Compared with the control group, dietary TH supplementation increased (*p* < 0.05) the relative abundances of microbial genes involved in the ubiquinone and other terpenoid quinone biosynthesis, styrene degradation, and biosynthesis of siderophore group nonribosomal peptides (Figure 9C). The relative abundances of microbial genes involved in the polycyclic aromatic hydrocarbon degradation and ascorbate/aldarate metabolism were enhanced (*p* < 0.05) in the LH-LF group compared with the control group (Figure 9D). Diets supplemented with LH-TH increased (*p* < 0.05) the relative abundances of microbial genes involved in the atrazine degradation and ethylbenzene degradation (Figure 9E). As shown in Figure 9F, dietary LF-TH supplementation increased (*p* < 0.05) the relative abundances of microbial genes involved in the ascorbate and aldarate metabolism compared with the control group. Compared with the control group, dietary LH-LF-TH supplementation increased (*p* < 0.05) the relative abundances of microbial genes involved in the retinol metabolism and aminobenzoate degradation (Figure 9G). Moreover, the relative abundances of microbial genes involved in the D-arginine and D-ornithine metabolism were increased (*p* < 0.05) in the LH and TH groups, whereas steroid biosynthesis, limonene and pinene degradation, and polyketide sugar unit biosynthesis were increased (*p* < 0.05) in the LH-LF, LH-TH, and LH-LF-TH groups, as well as the microbial genes involved in the ascorbate and aldarate metabolism and polyketide sugar unit biosynthesis in the TH and LF-TH groups.

### Correlation between microbiota abundances and morphology and physical barrier function of jejunum in laying hens

**Figure 10:**
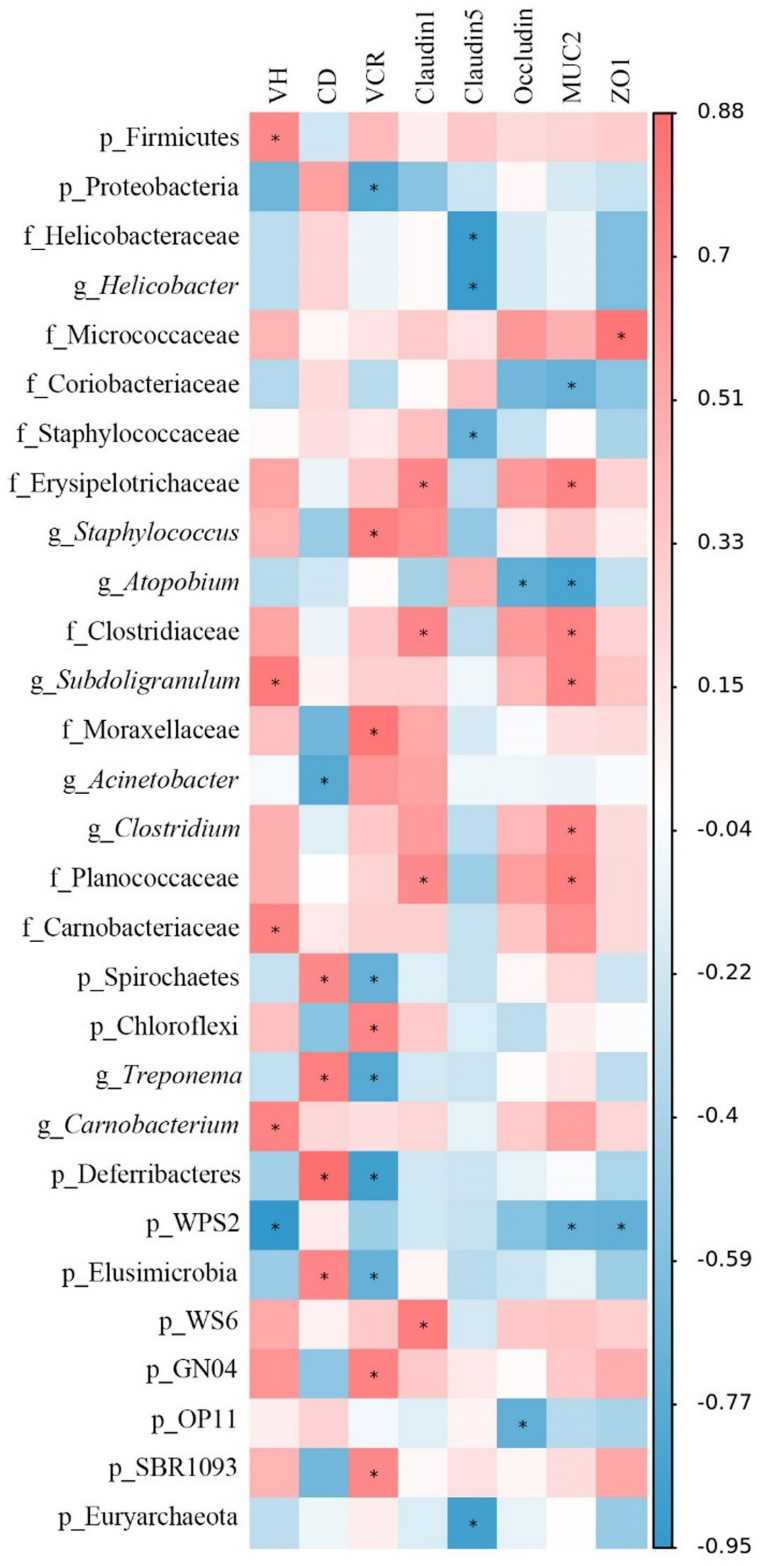
Spearman’s correlation analysis between jejunal morphology, gene expression level, and the microbiota abundances. Red represents a significant positive correlation, and blue represents a significant negative correlation. *p < 0.05. VH, villus height; CD, crypt depth; VCR, VH to CD ratio; *MUC-2*, mucin-2; *ZO-1*, zonula occludens-1.

The Spearman’s correlation analysis was performed to explore the differential abundances of jejunal microbiota associated with jejunal morphology and physical barrier function-related gene expressions (Figure 10). The VH was positively correlated (*p* < 0.05) with the relative abundances of *Firmicutes*, *Subdoligranulum*, *Carnobacteriaceae*, and *Carnobacterium*, while negatively (*p* < 0.05) correlated with *WPS2*. The CD was positively correlated (*p* < 0.05) with the relative abundances of *Spirochaetes*, *Treponema*, *Deferribacteres*, and *Elusimicrobia*, while negatively correlated (*p* < 0.05) with *Acinetobacter*. Moreover, the VCR was negatively correlated (*p* < 0.05) with the relative abundances of *Proteobacteria*, *Spirochaetes*, *Treponema*, *Deferribacteres*, and *Elusimicrobia*, whereas positively correlated (*p* < 0.05) with *Staphylococcus*, *Moraxellaceae*, *Chloroflexi*, *GN04*, and *SBR1093*. *Claudin-1* expression was positively correlated (*p* < 0.05) with the relative abundances of *Erysipelotrichaceae*, *Clostridiaceae*, *Planococcaceae*, and WS6. *Claudin-5* expression was negatively correlated (*p* < 0.05) with the relative abundances of *Helicobacteraceae*, *Helicobacter*, *Staphylococcaceae*, and *Euryarchaeota*. *Occludin* expression was negatively correlated (*p* < 0.05) with the relative abundances of *Atopobium* and *OP11*. *MUC2* expression was positively correlated (*p* < 0.05) with the relative abundances of *Erysipelotrichaceae*, *Clostridiaceae*, *Subdoligranulum*, *Clostridium*, and *Planococcaceae*, while negatively correlated (*p* < 0.05) with *Coriobacteriaceae*, *Atopbium*, and *WPS2*. Moreover, *ZO-1* expression was positively correlated (*p* < 0.05) with the relative abundance of *Micrococcaceae* and negatively correlated (*p* < 0.05) with *WPS2*.

## Discussion

During the late laying period, the laying performance, immune, and metabolic function of laying hens are decreased, and the egg quality is severely affected. Intestinal health is crucial to the health of the body and is closely related to the intestinal barrier function and microbiota. Therefore, the present study explored the effects of dietary CHUP supplementation on intestinal morphology, physical barrier functions, and microbial community composition in laying hens during the late laying period. The findings indicate that dietary CHUP improved the jejunal morphology and physical barrier function and increased the relative abundances of SCFAs and bacteriocins-producing bacteria and metabolic processes in the jejunum of laying hens.

The intestinal morphology reflects the intestinal function and health of animals (Liao and Nyachoti, 2017). The improvement of the bird’s nutrient absorption capacity mainly depends on the intestinal surface area, which is mainly associated with the intestinal mucosal structure (Gong et al., 2021). The intestinal epithelium is composed of abundant crypt-villus units. The absorptive surface area in villi could mediate nutrient transport (Clevers, 2013). In the present study, dietary LH-LF and LH-LF-TH supplementation increased the jejunal VCR, suggesting that these CHUP additives improved the intestinal nutrient absorption function of laying hens. A previous study demonstrated that dietary supplementation with leonurine hydrochloride at 120 mg/kg increased the VH and decreased the CD in the jejunum of 1-d-old male Ross broilers (Yang et al., 2019). However, dietary LH or TH had no impacts on the jejunal morphology of laying hens in the present study, probably because the combination compounds were more effective than the single Chinese herb. However, further studies are needed to elucidate the possible causes of such improvement.

Tight junction (TJ) proteins include claudins, occludin, and ZO-1 (Jian et al., 2015). Claudins play an indispensable role in maintaining the epidermal barrier (Furuse et al., 2002), and claudin-1 and claudin-5 are members of the claudins (Garcia-Hernandez et al., 2017). The down-regulation of *ZOs* and *claudins* (except *claudin-2*) expressions was found to be involved with the impairment of intestinal barrier function in the jejunal tissues of broilers (Yang et al., 2022). In the present study, dietary LH supplementation up-regulated the jejunal *claudin-1* expression, whereas dietary LF-TH up-regulated the *claudin-5* expression, suggesting that LH and LF-TH could improve the jejunal physical barrier function of laying hens. In addition, dietary CHUP supplementation down-regulated the jejunal *occludin* expression compared with the control group. Kuo et al. (2022) suggested that the down-regulation of *occludin* expression may be an adaptive response that limits tissue injury under inflammatory conditions. Furthermore, dietary LH, LF, and TH have anti-inflammatory characteristics, and the down-regulation of jejunal *occludin* expression might be one of the potential mechanisms of these Chinese herbs. The specific mechanism of these CHUP to improve the physical barrier of the intestine remains unclear and warrants further studies.

The intestinal microbiota is an important regulator of host metabolism (Tremaroli and Bäckhed, 2012). The correlation between microbiota composition or the relative abundances of bacterial species and physiological health has been previously reported (Hills et al., 2019). Previous studies reported that the improvement of feed conversion was related to the enhancement of intestinal bacteria diversity in laying hens (Zhou J. M. et al., 2021). In the present study, 16S rRNA sequencing results showed that dietary LH supplementation increased α-diversity indices of the jejunal microbiota compared to the LF-TH group. Moreover, the β-diversity analysis indicated significant separations between the control group and the LH-LF, LH-TH, and LH-LF-TH groups. These findings indicate that there were different effects on the richness and diversity of the intestinal microbiota between compounds and single Chinese herb, and the increased microbial richness in the jejunum might be beneficial for the growth of laying hens.

The intestinal microbiota, especially beneficial bacteria, regulates host health in various ways (Ding et al., 2021). Several intestinal bacteria, such as *Enterococcus*, *Bacteroides*, and *Feacalibacterium*, contribute to the body weight gain and egg-laying performance of hens (Han et al., 2022). *Blautia* is considered as a potential probiotic bacteria and is widely present in the intestines of mammals (Liu et al., 2021). In the present study, the relative abundances of *Blautia*, *Clostridia*, *Erysipelotrichaceae*, and *Carnobacterium* were enhanced by dietary LH supplementation. In recent years, metabolites produced by intestinal microbiota have attracted much attention due to their ability to regulate intestinal homeostasis (Sittipo et al., 2019). *Blautia* and *Carnobacterium* can produce bacteriocins with bactericidal activity and have non-bacteriolytic properties (Pilet et al., 1995). Bacteriocins are antimicrobial peptides that can inhibit pathogenic bacteria (Zou et al., 2018). Moreover, *Blautia*, *Clostridium* (Zhu et al., 2022), and *Erysipelotrichaceae* (Zhou J. et al., 2021) have been found to produce SCFAs, such as butyrate and propionate, which could improve the integrity of the intestinal epithelial and mucosal barrier. These bacteria species also positively affect the immune response and intestinal microbiota diversity (Peng et al., 2022). Collectively, these findings suggest that dietary LH may prevent the infection of antibiotic-resistant pathogens and would be beneficial for maintaining intestinal barrier integrity in laying hens. In addition, dietary LH-LF supplementation increased the abundances of several natural antimicrobial probiotics (i.e., *Enterococcus* and *Aeriscardovia*) and decreased some SCFAs-producing bacteria (i.e., *Feacalibacterium* and *Ruminococcus*). Research evidence reported that the active ingredients of Chinese herbs could be converted into bioactive metabolites after oral administration, which could affect intestinal microbiota composition (Lin et al., 2021). Therefore, the decreased SCFAs-producing bacteria may be related to the interaction of bioactive ingredients of the Chinese herbs with intestinal metabolites.

The interactions between intestinal microbiota and host nutrient metabolism have recently become a research hotspot (Zhang et al., 2020). Accumulating research evidence suggested that tetracycline, butirosin, and neomycin are important antibiotics that inhibit protein synthesis by binding to the bacterial ribosome (Kudo and Eguchi, 2009), and tetracycline has oxygen radical scavenging and anti-inflammatory properties (Griffin et al., 2010). In the present study, dietary LH supplementation enriched the abundances of microbial genes related to the biosynthesis of tetracycline, butirosin, and neomycin. This may be related to the increased bacteriocins-producing bacteria (*Blautia* and *Carnobacterium*) in the present study. In addition, the abundances of microbial genes related to the D-arginine and D-ornithine metabolism were enriched by dietary LH supplementation. Previous studies reported that D-arginine and D-ornithine metabolism in the intestine was associated with improved intestinal morphology and function by synthesizing polyamines and nitric oxide. Polyamines were found in the differentiation and proliferation of intestinal cells (Cynober, 1994). These findings indicate that the up-regulated *claudin-1* expression by dietary LH supplementation may be related to the regulation of D-arginine and D-ornithine metabolism in the jejunum of laying hens. Moreover, the present study also found that the abundances of jejunal microbial genes responsible for the limonene and pinene degradation were higher in the TH, LH-LF, LH-TH, and LH-LF-TH groups. Terpenoids are widely presented in Chinese herbs (Matkowski et al., 2013), and limonene and pinene are the intermediate products of terpenoids; suggesting that terpenoids present in Chinese herbs were effectively absorbed by laying hens after administration.

The Spearman’s correlation analysis revealed that jejunal *Erysipelotrichaceae*, *Clostridiaceae*, and *Planococcaceae* abundances were positively correlated with jejunal *claudin-1* expression. The positive correlation of these bacterial abundances may be explained by the fact that dietary LH up-regulated the jejunal *claudin-1* expression by increasing the abundance of jejunal SCFAs-producing bacteria. *Subdoligranulum* is a butyrate-producing bacteria (Qin et al., 2020). Zuo et al. (2020) reported that sodium butyrate could increase the VH and VCR in the small intestine of weaned lambs. The increased VH may be related to the production of sodium butyrate by the *Subdoligranulum*. These findings suggest that the effects of dietary LH and LH-LF-TH supplementation on jejunal morphology and physical barrier function may be partly responsible for the enrichment of potential probiotic bacteria species.

## Conclusion

In summary, dietary CHUP supplementation during the late laying period promoted jejunal physical barrier function in laying hens, which may be partly mediated by increasing the abundance of SCFAs and bacteriocins-producing bacteria and metabolic processes in the jejunum. Considering the beneficial effects of these CHUP on the jejunal morphology, gene expressions related to physical barrier function, and jejunal microbiota, dietary LH-LF, LF-TH, and LH-LF-TH supplementation had better effects, which could be potential dietary additives in poultry production.

## Data availability statement

The datasets presented in this study can be found in online repositories. The names of the repository/repositories and accession number(s) can be found at: https://www.scidb.cn/s/6VjuMz, 10.57760/sciencedb.07486.

## Ethics statement

The animal study was reviewed and approved by The Ethics Committee of the Institute of Subtropical Agriculture, Chinese Academy of Science.

## Author contributions

XK, YC, and JH designed the experiments. JG, WLi, CM, XH, and WLa conducted the experiments. JG, MA, and XK wrote and revised the paper. All authors contributed to the article and approved the submitted version.

## Funding

This study was supported by the City-School Cooperation Project of the Special Funds of Science and Technology in Fuyang City undertaken by Fuyang Normal University (SXHZ2020007) and the Special Funds of Construction of Innovative Provinces in Hunan Province (2019RS3022).

## Conflict of interest

The authors declare that the research was conducted in the absence of any commercial or financial relationships that could be construed as a potential conflict of interest.

## Correction note

A correction has been made to this article. Details can be found at: 10.3389/fmicb.2025.1628339.

## Publisher’s note

All claims expressed in this article are solely those of the authors and do not necessarily represent those of their affiliated organizations, or those of the publisher, the editors and the reviewers. Any product that may be evaluated in this article, or claim that may be made by its manufacturer, is not guaranteed or endorsed by the publisher.
