# Correction: Dietary supplementation with Chinese herb ultrafine powder improves intestinal morphology and physical barrier function by altering jejunal microbiota in laying hens

**DOI:** 10.3389/fmicb.2025.1628339

**Published:** 2025-06-09

**Authors:** Jue Gui, Md Abul Kalam Azad, Wenchao Lin, Chengwen Meng, Xin Hu, Yadong Cui, Wei Lan, Jianhua He, Xiangfeng Kong

**Affiliations:** ^1^Key Laboratory of Agro-ecological Processes in Subtropical Region, Hunan Provincial Key Laboratory of Animal Nutrition Physiology and Metabolic Processes, Institute of Subtropical Agriculture, Chinese Academy of Sciences, Changsha, Hunan, China; ^2^School of Biology and Food Engineering, Fuyang Normal University, Fuyang, Anhui, China; ^3^College of Animal Science and Technology, Hunan Agricultural University, Changsha, Hunan, China

**Keywords:** Chinese herb, intestinal barrier function, microbiota, ultrafine powder, Xinyang black-feather hens

In the published article, there was an error in the legend for Table 1 as published. In our study, due to an error, “The premix provided the following per kg of diets” should be revised to “The ingredients provided per kilogram of premix,” and “vitamin K, 3.18 g” should be revised to “vitamin K3, 0.18 g.” The corrected legend appears below.

^a^The ingredients provided per kilogram of premix: vitamin A, 140,000 IU; vitamin D3, 50,000 IU; vitamin E, 480 mg; vitamin K3, 0.18 g; vitamin B1, 63 mg; vitamin B2, 200 mg; vitamin B6, 140 mg; vitamin B12, 0.7 mg; nicotinic acid, 1000 mg; D-pantothenic acid, 500 mg; folic acid, 50 mg; D-biotin, 5.0 mg; choline chloride, 900 mg; Fe, 2.0 g; Cu, 0.3 g; Mn, 1.8 g; Zn, 2.0 g; I, 70 mg; Se, 9 mg; and phytase, 3000 IU.

^b^Crude protein and ether extract were measured values, while the others were calculated values.

The authors apologize for this error and state that this does not change the scientific conclusions of the article in any way. The original article has been updated.

