# Supplementary material for: Dietary supplementation with Chinese herb ultrafine powder improves intestinal morphology and physical barrier function by altering jejunal microbiota in laying hens
Source: Front Microbiol. 2023 May 16;14:1185806. doi: 10.3389/fmicb.2023.1185806 (PMC10227515; doi:10.3389/fmicb.2023.1185806)
Supplement: Supplementary file 1 [file Table_1.DOCX]

Supplementary Material

Dietary supplementation with Chinese herb ultrafine powder improves intestinal morphology and physical barrier function by altering jejunal microbiota in laying hens

**Jue Gui*, Md. Abul Kalam Azad, Wenchao Lin, Chengwen Meng, Xin Hu, Yadong Cui, Wei Lan, Jianhua He,** **Xiangfeng Kong**

*** Correspondence:** Xiangfeng Kong: [nnkxf@isa.ac.cn](mailto:nnkxf@isa.ac.cn)

# Supplementary Data

**Table 1** Primer sequences used for real-time PCR.

| Genes  name | Primer sequence (5′-3′) | Accession No. | Product  Size  (bp) |
| --- | --- | --- | --- |
| *Claudin-1* | F: GGGTCTGGTTGGTGTGTTTG  R: TCTGGTGTTAACGGGTGTGA | NM_001013611.2 | 205 |
| *Claudin-5* | F: CATCACTTCTCCTTCGTCAGC  R: GCACAAAGATCTCCCAGGTC | NM_204201.2 | 111 |
| *Occludin* | F: ACAGCCCTCAATACCAGGATGTG  R: ACCATGCGCTTGATGTGGAA | XM_046904540.1 | 133 |
| *MUC-2* | F: GCTACAGGATCTGCCTTTGC  R: AATGGGCCCTCTGAGTTTTT | XM_040673077.2 | 152 |
| *ZO-1* | F: TATAGAAGATCGTGCGCCTCC  R: GAGGTCTGCCATCGTAGCTC | XM_046925214.1 | 209 |
| *β-actin* | F: AGTACCCCATTGAACACGGT  R: ATACATGGCTGGGGTGTTGA | NM_205518.2 | 197 |

*MUC-2*, mucin-2; *ZO-1*, zona occludens-1.
